# Tribolium castaneum defensin 1 kills Moraxella catarrhalisin an in vitro infection model but does not harm commensal bacteria

**DOI:** 10.1080/21505594.2021.1908741

**Published:** 2021-04-12

**Authors:** Wilhelm Bertrams, Nora S. Lindhauer, Marie Christin Rieke, Anne Paas, Kerstin Hoffmann, Brandon Greene, Alexander Visekruna, Andreas Vilcinskas, Kerstin Seidel, Bernd Schmeck

**Affiliations:** a Institute for Lung Research, Universities of Giessen and Marburg Lung Center, Philipps-University Marburg, Marburg, Germany; b Department of Bioresources, Fraunhofer Institute for Molecular Biology and Applied Ecology, Giessen, Germany; c Institute of Medical Bioinformatics and Biostatistics, Universities of Giessen and Marburg, Philipps-University Marburg, Marburg, Germany; d Institute for Medical Microbiology and Hospital Hygiene, Philipps University of Marburg, Germany; e Institute for Insect Biotechnology, Justus-Liebig-University, Giessen, Germany; f Department of Medicine, Pulmonary and Critical Care Medicine, University Medical Center Giessen and Marburg, Philipps-University Marburg, Marburg, Germany; g Member for Infectious Diseases (DZIF), and the SYNMIKRO Center for Synthetic Microbiology, Philipps-University Marburg, Marburg, Germany; h Institute for Lung Health (ILH), Giessen, Germany

**Keywords:** Antimicrobial peptides, *Moraxella catarrhalis*, macrophages, inflammation, insect, antibiotic resistance, defensin, commensals

## Abstract

*Moraxella catarrhalis* is a bacterial pathogen that causes respiratory tract infections in humans. The increasing prevalence of antibiotic-resistant *M. catarrhalis* strains has created a demand for alternative treatment options. We therefore tested 23 insect antimicrobial peptides (AMPs) for their activity against *M. catarrhalis* in a human *in vitro* infection model with primary macrophages, and against commensal bacteria. Effects on bacterial growth were determined by colony counting and growth curve analysis. The inflammatory macrophage response was characterized by qPCR and multiplex ELISA. Eleven of the AMPs were active against *M. catarrhalis*. Defensin 1 from the red flour beetle *Tribolium castaneum* significantly inhibited bacterial growth and reduced the number of colony forming units. This AMP also showed antibacterial activity in the *in vitro* infection model, reducing cytokine expression and release by macrophages. Defensin 1 had no effect on the commensal bacteria *Escherichia coli* and *Enterococcus faecalis*. However, sarcotoxin 1 C from the green bottle fly *Lucilia sericata* was active against *M. catarrhalis* and *E. coli*, but not against *E. faecalis*. The ability of *T. castaneum* defensin 1 to inhibit *M. catarrhalis* but not selected commensal bacteria, and the absence of cytotoxic or inflammatory effects against human blood-derived macrophages, suggests this AMP may be suitable for development as a new therapeutic lead against antibiotic-resistant *M. catarrhalis*.

## Introduction

*Moraxella catarrhalis* is a Gram-negative, aerobic, diplococcus pathogen that colonizes the human respiratory tract. It produces non-hemolytic, round and opaque colonies on blood agar. The major virulence strategies of *M. catarrhalis* include complement resistance, the formation of protective bioﬁlms, localization within lymphoid tissues to avoid immunosurveillance, and polyclonal non-speciﬁc B-cell activation to modulate adaptive immunity [1]. Respiratory tract colonization does not always lead to symptomatic disease, which is highly dependent on the patient’s age [2]. In the upper respiratory tract, *M. catarrhalis* can cause acute otitis media (OM). Approximately 80% of children have already experienced OM by the age of 3, and 15–20% of these cases are caused by *M. catarrhalis*. The prevalence of *M. catarrhalis* is ≤ 75% in children but only 1–3% in adults, a distribution that has been stable since the 1970s [3]. Infections with *M. catarrhalis* can be treated with antibiotics, but strains resistant to penicillin, ampicillin, and amoxicillin are now common [4]. Antibiotic-resistant Gram-negative bacteria are increasingly seen as threats to global healthcare systems, causing more than 670,000 infections and 33,000 deaths per year in Europe [5] with associated healthcare costs of more than €1 billion [6]. Alternative treatments for bacterial infections are therefore urgently needed.

Antimicrobial peptides (AMPs) offer one potential source of new drug leads against infectious diseases. They are short peptides (typically 12–50 amino acids) that function as part of the innate immune system in all eukaryotic organisms. Their spectrum of activity includes viruses, bacteria, fungi and parasites. Insects provide a source of particularly diverse AMPs, which can be classified on the basis of their chemical attributes [7]. AMPs demonstrate a broad range of cellular mechanisms, from the promotion of angiogenesis to host cell chemotaxis, but electrostatic interaction with bacterial membranes and subsequent pore formation is the most important mechanism of direct bactericidal action [8]. In this regard, we previously described the effect of defensin 1 from the red flour beetle (*Tribolium castaneum*) against *Streptococcus pneumoniae* [7].

In this study, we tested 23 insect AMPs for their activity against *M. catarrhalis* and selected two with the most promising activity for further analysis in an *in vitro* infection model based on primary human macrophages. We selected *T. castaneum* defensin 1, a β-sheet globular AMP stabilized by intramolecular disulfide bridges, and sarcotoxin 1 C from the green bottle fly *Lucilia sericata* [9], a linear α-helical AMP without cysteine residues. We investigated the ability of these AMPs to trigger cytokine release, host cell cytotoxicity, hemolysis, inflammation and immunosuppression. Our results may facilitate the development of AMP-based drug leads against antibiotic-resistant Gram-negative bacteria including *M. catarrhalis.*

## Materials and methods

### Antimicrobial peptides

The 23 insect-derived AMPs tested in this study were produced by solid-phase synthesis and purified by Coring System Diagnostix (Gernsheim, Germany), GenScript (Piscataway, NJ, USA) and Pepmic (Suzhou, China). The integrity of the AMPs was confirmed by liquid chromatography-mass spectrometry. The properties of the AMPs are summarized in Table S1, chromatograms for each peptide are provided in Table S2.

## Culture and growth kinetics of *M. catarrhalis*

Colonies of *M. catarrhalis* were grown on sheep blood agar plates for 12 h at 37°C and 5% CO_2_ before transfer to brain heart infusion (BHI) medium (Carl Roth, Karlsruhe, Germany) at an initial concentration of 1.6 × 10^7^ cells/ml (OD_600_ = 0.08) as determined using an Ultraspec 10 cell densitometer (Amersham BioSciences, Little Chalfont, UK). Bacteria were cultivated in a shaking incubator at 37°C until the concentration reached 1.2 × 10^8^ cells/ml (OD_600_ = 0.6) and then diluted to 2 × 10^6^ cells/ml (OD_600_ = 0.011). To establish the optimal concentration ranges for AMP activity, defensin 1 was prepared as a two-fold dilution series from 12.5 µM to 1.56 µM and was added to the bacterial cultures, which were incubated as above for a further 16 h. Untreated cultures were used as controls. The OD_600_ was measured automatically at 30-min intervals using an Infinite M200 Pro plate reader (Tecan Life Sciences, Männedorf, Switzerland). For AMP inhibitory testing without macrophages, *M. catarrhalis* was grown to the mid-exponential phase (~5 x 10^8^ cells/ml) in BHI medium in the presence of defensin 1 (12.5 µM) or sarcotoxin 1 C (0.39 µM) before dilution in PBS containing 0.15% gelatin, and directly plated on sheep blood agar. They were cultivated for a further 15 h at 37°C and 5% CO_2_ in serial dilution before manual counting of the colonies on each plate.

## Culture and growth kinetics of *E. faecalis* and *E. coli*

For the analysis of commensal bacteria, *E. faecalis* was grown on Columbia agar plates or in liquid BHI medium, and *E. coli* was grown on McConkey agar or in liquid LB medium. After overnight culture on agar plates at 37°C and 5% CO_2_, both species were transferred to liquid medium at an initial OD_600_ of 0.005. The cultures were maintained in a shaking incubator at 37°C, and the OD_600_ was measured automatically at 30-min intervals as above.

## Colony forming unit assay

The absolute number of bacterial cells after treatment with defensin 1 or sarcotoxin 1 C was determined by counting the colony forming units (CFUs). *M. catarrhalis* overnight cultures prepared as described above were grown to the mid-exponential phase (~5 x 10^8^ cells/ml) in BHI medium before dilution to 2 × 10^7^ cells/ml in PBS containing 0.15% gelatin, and blood-derived macrophages (BDMs) were infected with a multiplicity of infection (MOI) of 0.5 or 1. After incubation with bacteria for 1 or 5 h, we added 12.5 µM defensin 1 or 0.39 µM sarcotoxin 1 C to the cell cultures representing each incubation time point, based on the concentrations previously shown to inhibit bacterial replication. Cells were then incubated for an additional 16 h at 37°C and 5% CO_2_ and then lysed with 1% saponin. Lysates were plated on sheep blood agar and cultivated for a further 15 h at 37°C and 5% CO_2_ before manual counting of the colonies on each plate.

## BDM cultivation and differentiation

All donors gave informed written consent (Ethics approval number: 161/17). Human BDMs were cultured as previously described [7]. Briefly, primary human monocytes were isolated from donor buffy coats by selection for CD14^+^ cells using CD14 Microbeads (Miltenyi Biotec, Bergisch Gladbach, Germany). Monocytes were grown in RPMI1640 medium with 1% human AB serum at 37°C and 5% CO_2_ on ultra-low attachment plates (Sigma-Aldrich, Munich, Germany). After 6 days, differentiated macrophages were detached, seeded at the desired density and incubated for 24 h before further analysis.

## Isolation of RNA from infected BDMs and real-time PCR analysis

BDMs were infected with *M. catarrhalis* as described above and the cells and supernatant were collected 16 h post-infection. Total RNA was isolated by phenol-chloroform extraction followed by reverse transcription using the High-Capacity RNA-to-cDNA kit (Thermo Fisher Scientific, Waltham, MA, USA). Quantitative real-time PCR (qRT-PCR) was performed with the following specific primer pairs to measure the expression of IL-1β (forward primer 5′-AGC TCG CCA GTG AAA TGA TGG-3′ and reverse primer 5′-CAG GTC CTG GAA GGA GCA CTT C-3′), IL-8 (forward primer 5′-ACT GAG AGT GAT TGA GAG TGG AC-3′ and reverse primer 5′-AAC CCT CTG CAC CCA GTT TTC-3′), and RPS18 (forward primer 5′-GCG GCG GAA AAT AGC CTT TG-3′ and reverse primer 5′-GAT CAC ACG TCC ACC TCA TC-3′).

## Multiplex ELISA

The presence of cytokines in the BDM supernatant after infection and treatment with 12.5 µM defensin 1 was assessed using the MAGPIX Multiplex ELISA (Luminex, Austin, TX, USA). BDM supernatants were prepared as recommended for the MAGPIX system. The cytokine panel comprised MIP1-α, MCP1, IL-1β, IL-6, IL-8, IL-10, IL-12p70, IL-23, LAP and TNF-α.

## Statistical analysis

Statistical interpretation of the multiplex ELISA data required the incorporation of data points outside the detection range and adjustments for the effect of parallel technical measurements from the same biological samples. We therefore used the Hodges and Lehmann nonparametric aligned ranks test [10] to compare cytokine secretion by treatment time within treatment groups and between the following control and treatment groups:

MOI = 0.5 without defensin 1 (control)

MOI = 0.5 with defensin 1 given 1 h post-infection

MOI = 0.5 with defensin 1 given 5 h post-infection

MOI = 1 without defensin 1 (control)

MOI = 1 with defensin 1 given 1 h post-infection

MOI = 1 with defensin 1 given 5 h post-infection

LPS without defensin 1

LPS plus defensin 1 (added 1 h after LPS)

Statistical analysis was carried out using the R suite. All other statistical tests were carried out as indicated in the figure legends, with a significance threshold of p < 0.05.

## Results

### *Defensin 1 and sarcotoxin 1 C show efficacy against* M. catarrhalis

**Figure 1.**
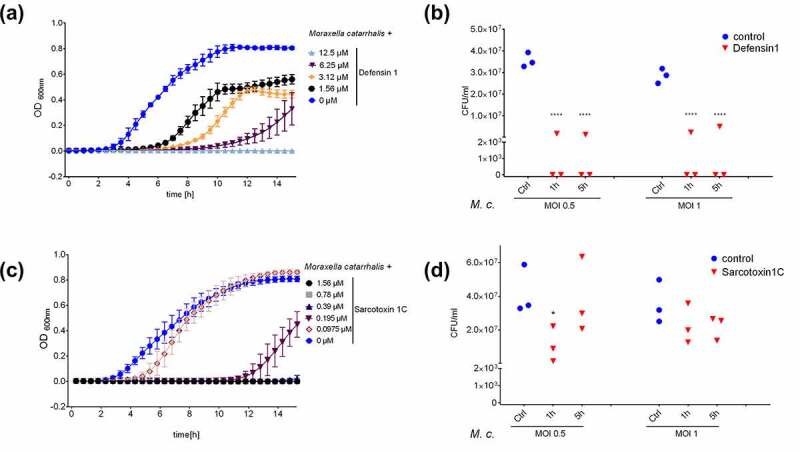
**Defensin 1 and sarcotoxin 1 C inhibit the growth of *M. catarrhalis***. Bacteria were grown to OD_600_ = 0.6 in BHI medium, diluted to OD_600_ = 0.011 and incubated with two-fold serial dilutions of defensin 1 (between 25 µM and 0 µM) or sarcotoxin 1 (between 1.56 µM and 0 µM) at 37°C. The OD_600 nm_ was measured at 30-min intervals (a and c). Bacterial growth was also monitored following the infection of BDMs at the indicated MOIs, with AMP treatment beginning 1 or 5 h post-infection (defensin 1 = 12.5 µM, sarcotoxin 1 C = 0.39 µM, with uninfected cells as controls) (b and d). Statistical significance was assessed by two-way ANOVA (****p < 0.0001 vs. control)

We screened a panel of 23 AMPs, 11 of which were effective against *M. catarrhalis* (Table S1). Among those, we selected defensin 1 and sarcotoxin 1 C for further analysis because they differ in structure and hence in their potential mechanism of action. Defensin 1 completely abolished the growth of *M. catarrhalis* when present at a concentration of 12.5 µM, but only delayed growth at a concentration of 6.25 µM (Figure 1a). Defensin 1 also reduced the bacterial burden in an infection assay of primary BDMs, as reflected by the lower *M. catarrhalis* CFU count compared to assays without the peptide (Figure 1b). Sarcotoxin 1 C completely inhibited the growth of *M. catarrhalis* at a concentration of 0.39 µM and caused a growth delay at 0.195 µM (Figure 1c). Sarcotoxin 1 C also reduced the bacterial load in an infection assay of primary BDMs when presented at a concentration of 0.39 µM (Figure 1d). While defensin 1 efficiently killed the bacteria, the effect of sarcotoxin 1 C on bacterial growth was partly reversible upon dilution of the AMP by plating (Figure S1).

### *Defensin 1 reduces the survival of* M. catarrhalis *and the inflammatory activation of macrophages*

**Figure 2.**
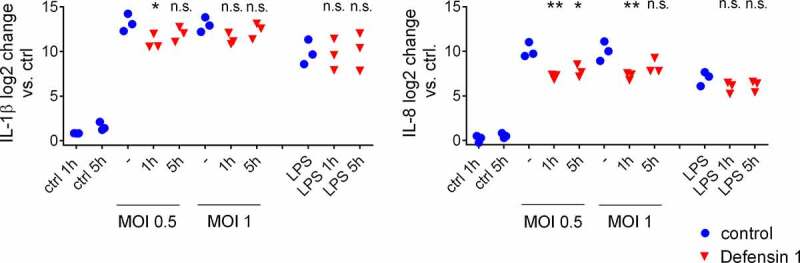
**Defensin 1 reduces the abundance of cytokine mRNAs in BDMs infected with *M. catarrhalis***. Treatment with 12.5 µM defensin 1 (minimal inhibitory concentration) significantly reduced the amount of *IL-1β* mRNA (a) and *IL-8* mRNA (b) expression for all MOIs and time points. LPS was used as a sterile positive control. Log_2_ transformed data are shown. Statistical significance was assessed by two-way ANOVA (**p < 0.01, *p < 0.05)

**Figure 3.**
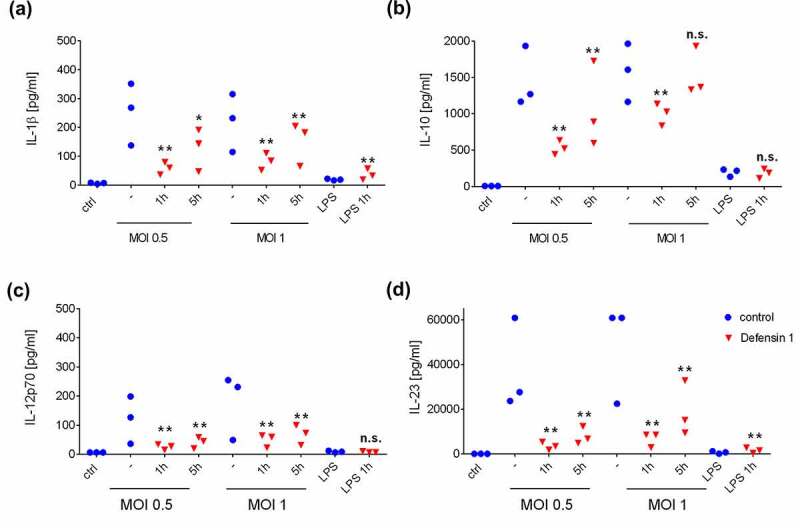
Defensin 1 inhibits the secretion of selected cytokines from human macrophages infected with *M. catarrhalis.*

Defensin 1 showed efficacy in the CFU and infection assays, but for therapeutic development it must also demonstrate minimal toxicity toward human cells. We therefore tested the ability of defensin 1 to induce inflammation in an *in vitro* infection assay with BDMs. Previous work has shown that defensin 1 has minimal hemolytic activity up to a concentration of 100 µM and is nontoxic toward macrophages [7]. We exposed BDMs to defensin 1 at a concentration of 12.5 µM 1 or 5 h after infection with *M. catarrhalis* at MOI = 0.5 or MOI = 1 to mimic a clinical setting. Defensin 1 limited the expression of IL-1β and IL-8 at both post-infection time points and both MOI values, as determined by qRT-PCR (Figure 2). Defensin 1 also inhibited the bacteria-induced secretion of key cytokines IL-1β, IL-10, IL-12p70 and IL-23 at both post-infection time points and both MOI values, as determined by multiplex ELISA (Figure 3). The sterile activation of BDMs by LPS was not significantly affected by the presence of defensin 1, as previously also reported for TNF-α [7].

Supernatants were analyzed by multiplex ELISA. The quantity of secreted IL-1β (a), IL-10 (b), IL-12p70 (c) and IL-23 (d) was measured following treatment with defensin 1 (12.5 µM) 1 or 5 h post-infection. LPS was used as a sterile positive control and uninfected cells as a negative control. Statistical significance was determined as stated in the methods section (*p < 0.05 and **p < 0.01 vs. corresponding sample without AMP).

### *Commensal* E. faecalis *and* E. coli *are unaffected by defensin 1 and only partly affected by sarcotoxin 1 C*

**Figure 4.**
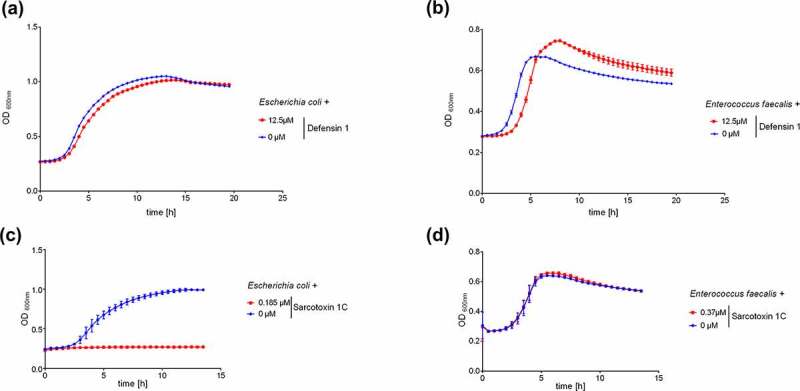
Defensin 1 and sarcotoxin 1 C differ in their effect against two commensal bacteria

Antibiotics often cause severe clinical side effects by disrupting the commensal flora [11]. We therefore investigated the impact of defensin 1 on the commensal bacteria *E. faecalis* and *E. coli*. Whereas 12.5 µM defensin 1 was sufficient to completely inhibit the growth of *M. catarrhalis*, the same concentration only caused a minor growth delay in both commensal species (Figure 4a and b). Interestingly, 0.39 µM sarcotoxin 1 C was able to inhibit the growth of *E. coli* but not *E. faecalis* (Figure 4c and d).

*E. faecalis* was grown on Columbia blood agar plates and *E. coli* was grown on McConkey agar plates overnight. The bacteria were then transferred to liquid medium (OD_600_ = 0.005) and incubated with defensin 1 (a and b) or sarcotoxin 1 C (c and d) at 37°C. The OD_600_ was measured at 30-min intervals. Three biological replicates are shown with mean values ± standard deviations. Defensin 1 (12.5 µM) showed negligible activity against both species, whereas sarcotoxin 1 C (0.39 µM) selectively inhibited the growth of *E. coli*.

## Discussion

Insects produce a broad repertoire of AMPs offering a rich source of potential new drug leads for the treatment of infections caused by antibiotic-resistant bacteria. We therefore screened a panel of 23 previously-described insect AMPs [7] against *M. catarrhalis*, a pathogen that causes respiratory infections in humans and is considered an emerging threat due to the increasing prevalence of antibiotic-resistant strains [4]. The most effective AMP in the panel was *T. castaneum* defensin 1, which was previously found to show low toxicity toward human BDMs [7]. Here we found that defensin 1 reduced the CFU counts of *M. catarrhalis* without harming BDMs in our *in vitro* infection model. It also limited the induction of the *IL-8* and *IL-1β* genes compared to untreated BDMs, and inhibited the secretion of cytokines such as IL-23, IL-12p70, IL-1β and IL-10. Despite its potent activity against *M. catarrhalis*, defensin 1 had a negligible impact on the growth of the commensal bacteria *E. faecalis* and *E. coli*. Another AMP in our panel with activity against *M. catarrhalis* was *L. sericata* sarcotoxin 1 C. This AMP was also effective against *E. coli*, but not against *E. faecalis*.

AMPs exert their function by targeting the bacterial cell wall, and are usually specialized for either Gram-negative or Gram-positive bacteria. Many AMPs are amphipathic with a positive net charge, so they bind to the negatively charged components of the bacterial cell wall and outer membrane. Some AMPs form pores in the bacterial cell membrane by direct binding and integration, leading to lysis and cell death [12]. Because AMPs target the structural integrity of bacteria, it is more difficult for them to evolve resistance mechanisms. The unique potency of defensin 1 against *M. catarrhalis* may reflect the presence of multiple disulfide bonds that help to stabilize the peptide and prevent degradation [13]. The β-sheet secondary structure may also increase stability by preventing the conformational changes that occur in α-helical AMPs. The enhanced stability of defensin 1 may explain its ability to inhibit the growth of *M. catarrhalis* in the BDM infection model, thus reducing the inflammatory host response triggered by bacterial cells. The timing of defensin 1 administration 1 or 5 h post-infection was chosen to mimic the clinical environment, where antimicrobial treatment tends to be initiated shortly after infection. We found that defensin 1 treatment 1 h post-infection was slightly more eﬀective than the treatment after 5 h, which we attribute to a more comprehensive establishment of infection and more robust bacterial growth at the 5 h time point.

The approval of AMPs for the treatment of severe infections is pending [14], but it is important to note that AMPs not only act as potent direct antimicrobial agents but also as immunomodulators, thus helping to marshal the immune system against invading pathogens [15]. For example, AMPs can influence immune cell diﬀerentiation, the stimulation of chemotaxis, anti-endotoxin activity, initiation of adaptive immunity, and suppression of the TLR-mediated production of cytokines [16–18].

Among the 23 AMPs we tested, only defensin 1 was previously found to be effective against the Gram-positive pathogen *Streptococcus pneumoniae* [7]. It is unclear whether the eﬃcacy of defensin 1 indeed lies in the structure of the peptide and whether other AMPs with different structures would also show activity against *M. catarrhalis*. We therefore also investigated *L. sericata* sarcotoxin 1 C, which has a linear α-helical structure without disulfide bridges. This AMP was efficient at a concentration of 0.39 µM, making it considerably more potent than defensin 1 in stopping bacterial growth in solution.

Given their different chemical structures and the different efficacies of defensin 1 and sarcotoxin 1 against *M. catarrhalis*, we tested whether their capacity to inhibit growth was bactericidal or bacteriostatic. While defensin 1 completely prevented bacterial growth also after its dilution, *M. catarrhalis* resumed growth after treatment with sarcotoxin 1 C. This suggests that defensin 1 has bactericidal capacity, while the effect of sarcotoxin 1 C is primarily bacteriostatic, as has been described before for the respective molecule class [19,20].

We furthermore tested the effect of defensin 1 and sarcotoxin 1 C on the commensals *E. faecalis* and *E. coli*. Selectivity of AMPs may provide an important therapeutic advantage over broad-spectrum antibiotics because AMPs targeting pathogens but not commensals would avoid the common side effects of antibiotic therapy. Intriguingly, whereas sarcotoxin 1 C efficiently inhibited the growth of Gram-negative *E. coli* as previously reported [21], it showed no activity against Gram-positive *E. faecalis*. Sarcotoxin 1 C was previously shown to inhibit 90% of clinical multidrug-resistant isolates of *Enterobacter cloacae, Acinetobacter baumannii* and *Salmonella enterica*, and pharmacological profiling revealed a good *in vitro* therapeutic index, no cytotoxicity or cardiotoxicity, an inconspicuous broad-panel off-target profile, and no acute toxicity in mice at a dose of 10 mg/kg [21]. Defensin 1 had had no effect against either commensal species in a previous study [20]. Cationic murine α-defensins, which have a predominantly β-sheet secondary structure and contain disulfide bonds, have been shown to kill *E. coli* [22]. Sarcotoxin 1 C and defensin 1 are cationic AMPs, allowing them to bind the outer membrane of Gram-negative bacteria and the cell wall of Gram-positive bacteria [23]. More mechanistic data are required to shed light on the selective action of the AMPs we describe and their association with different bacterial membrane and cell wall structures.

In summary, we have characterized the activity of defensin 1 against *M. catarrhalis* and confirmed its negligible activity against the commensals *E. faecalis* and *E. coli*. In contrast, we found that sarcotoxin 1 C was active against *M. catarrhalis* and *E. coli*, but ineffective against *E. faecalis*. We conclude that both sarcotoxin 1 C and defensin 1 are promising leads for the development of new antibiotics against *M. catarrhalis* infections, and that defensin 1 is particularly suitable due to its negligible effect against selected commensal flora. While the effects of defensin 1 and sarcotoxin have partly been described before, the strength of our study lies in the direct comparison of their action against commensal and pathologic bacteria. With *M. catarrhalis*, we chose an important pathogen with potentially chronic disease manifestation, which we show for the first time to be sensitive to defensin 1 and sarcotoxin 1 C. The clinical implications of AMPs against pathogens of the airways necessitates development of topical application of AMPs to the lung epithelium, which requires the large-scale production of AMPs as aerosol formulations [24]. Recently, successful attempts have been made to neutralize *Pseudomonas aeruginosa* with AMPs coupled to nanoparticles in a mouse model [25], and also to render AMPs inhalable by spray-drying [26]. In the advent of spreading antibiotics resistance, AMPs hold great potential as successors or support of classical antibiotic treatment.

## Supplementary Material

Supplemental Material
