# Supplementary material for: Tribolium castaneum defensin 1 kills Moraxella catarrhalisin an in vitro infection model but does not harm commensal bacteria
Source: Virulence. 2021 Apr 12;12(1):1003–10. doi: 10.1080/21505594.2021.1908741 (PMC8043168; doi:10.1080/21505594.2021.1908741)
Supplement: Supplemental Material [file KVIR_A_1908741_SM1272.zip › Document.rtf]

Figure S1: 
M. catarrhalis was grown to the mid-exponential phase (~5 x 10^8^ cells/ml) in BHI medium in the presence of defensin 1 (12.5 µM) or sarcotoxin 1C (0.39 µM) before dilution in PBS containing 0.15% gelatin, and directly plated on sheep blood agar. They were cultivated for a further 15 h at 37 °C and 5% CO2 in serial dilution before manual counting of the colonies on each plate. 
