# Supplementary material for: Tribolium castaneum defensin 1 kills Moraxella catarrhalisin an in vitro infection model but does not harm commensal bacteria
Source: Virulence. 2021 Apr 12;12(1):1003–10. doi: 10.1080/21505594.2021.1908741 (PMC8043168; doi:10.1080/21505594.2021.1908741)
Supplement: Supplemental Material [file KVIR_A_1908741_SM1272.zip › TableS1.pdf]

Table S1. Detailed characterization of tested AMPs. *M.c.* were grown to an Optical Density (OD) 600nm of 0.6 in Brain Heart Infusion (BHI) Medium.
Upon dilution to OD_{600nm}= 0.01, *M.c.* were incubated with declining amounts of indicated AMPs (25 µM to 0.048 µM at halving intervals) at 37°C with 5% CO_2.
OD600nm was measured at 30 min intervals to establish the minimal inhibitory concentration (MIC). pI = isoelectric point, MW = molecular weight in kiloDalton.

MIC	AMP name	AMP class	Organism	Amino Acid Sequence	pI	MW (kDa)	Purity (%)	Retention time [min]	modification
**0.195 µM**	Cecropin A	α-helical	*Hyalophora cecropia*	KWKLFKKIEKVGQNIRDGIIKAGPAVAVVGQATQIAK	10.75	4.00	91.06	7.30	n-term. amidation
**0.39 µM**	Sarcotoxin IA	α-helical	*Sarcophaga peregrina*	GWLKKIGKKIERVGQHTRDATIQGLGIAQQAANVAATAR	11.07	4.16	91.37	9.37	n-term. amidation
**1. 56µM**	Cecropin A	α-helical	*Aedes aegypti*	GGLKKLGKKLEGAGKRVFNAAEKALPVVAGAKALRK	10.79	3.67	90.38	11.76	none
**>25µM**	Ceratotoxin		*Ceratitis capitata*	SIGSAFKKALPVAKKIGKAALPIAKAALP	10.70	2.89	93.60	9.38	none
**~ 25µM**	Stomoxyn	α-helical	*Stomoxys calcitrans*	RGFRKHFNKLVKKVKHTISETAHVAKDTAVIAGSGAAVVAAT	11.26	4.40	95.34	10.08	n-term. amidation
**~ 25µM**	Spinigerin	linear	*Pseudacanthotermes spiniger*	HVDKKVADKVLLLKQLRIMRLLTRL	11.07	3.00	96.23	12.45	none
**>25µM**	Apidaecin Ia	proline-rich	*Apis mellifera*	GNNRPVYIPQPRPPHPRI	11.71	2.11	92.98	16.91	none
**>25µM**	Apidaecin	proline-rich	*Bombus pascuorum*	GNRPVYIPPPRPPHPRL	11.71	2.00	92.03	16.36	none
**>25µM**	Formaecin-1	proline-rich	*Myrmecia gulosa*	GRPNPVNNKPTPHPRL	12.01	1.80	93.53	13.93	none
**>25µM**	Drosocin	proline-rich	*Drosophila melanogaster*	GKPRPYSPRPTSHPRPIRV	12.01	2.20	92.86	11.65	none
**>25µM**	Pyrrhocoricin	proline-rich	*Pyrrhocoris apterus*	VDKGSYLPRPTPPRPIYNRN	10.27	2.34	93.17	11.38	none
**>25 µM**	Metalnikowin-1	proline-rich	*Palomena prasina*	VDKPDYRPRPRPPNM	9.98	1.84	92.85	13.36	none
**>25 µM**	Metalnikowin-2A	proline-rich	*Palomena prasina*	VDKPDYRPRPWPRPN	9.98	1.84	92.39	10.85	none
**>25 µM**	Lebocin-1	proline-rich	*Bombyx mori*	DLRFLYPRGKLPVPTPPPFNPKPIYIDMGNRY	9.82	3.77	92.58	12.32	none
**>25 µM**	Abaecin	proline-rich	*Bombus pascuorum*	FVPYNPPRPGQSKPFPSFPGHGPFNPKIQWPYPLPNPGH	10.00	4.39	90.07	12.03	none
**>25 µM**	Metchnikowin-1	proline-rich	*Drosophila melanogaster*	HRHQGPIFDTRPSPFNPNQPRPGPIY	10.74	3.03	99.06	11.50	none
**>25 µM**	Metchnikowin-2	proline-rich	*Drolophila melanogaster*	HRRQGPIFDTRPSPFNPNQPRPGPIY	11.54	3.04	97.16	11.46	none
**12.5 µM**	Defensin 1	disulfide stabilized	*Tribolium castaneum*	VTCDLLSAEAKGVKVNHAACAAHCLLKRKRGGYCNKRRICVCRN	9.7	4.83	90.53	18.41	none
**~ 25 µM**	Cecropin-like	α-helical	*Lucilia sericata*	HHLFGKVGREIERSAHKVGHKLEHVRHEVSKTAKKVDKVVGHIK TAKKVVAAAGAIAGVVAAA	10.35	6.66	90.28	12.01	none
**0.195 µM**	Cecropin	α-helical	*Galleria mellonella*	KWKIFKKIEKAGRNIRDGIIKAGPAVSVVGEAATIYKTG	10.21	4.21	93.61	12.33	n-term. amidation
**0.37 µM**	Cecropin (Sarcotoxin 1C)	α-helical	*Lucilia sericata*	GWLKKIGKKIERVGQHTRDATIQTIGVAQQAANVAATLKG	10.56	4.26	94.31	10.83	n-term. amidation
**12.5 µM**	Stomoxyn	α-helical	*Lucilia sericata*	GFRKRFNKLVKKVKHTIKETANVSKDVAIVAGSGVAVGAAMG	10.73	4.38	90.37	13.35	n-term. amidation n-term. amidation
**1.56 µM**	Cecropin (Sarcotoxin)	α-helical	*Eristalis tenax*	GFLKKIGKKLEGAVQRTRDATIQTIAVAQAAANVAATAKQ	10.56	4.14	90.80	9.76	none
