# Supplementary material for: Tribolium castaneum defensin 1 kills Moraxella catarrhalisin an in vitro infection model but does not harm commensal bacteria
Source: Virulence. 2021 Apr 12;12(1):1003–10. doi: 10.1080/21505594.2021.1908741 (PMC8043168; doi:10.1080/21505594.2021.1908741)
Supplement: Supplemental Material [file KVIR_A_1908741_SM1272.zip › tabs2.pdf]

Column :4.6*250mm, phenomenex C18-5
Solvent A :0.1%Trifluoroacetic in 100% Acetonitrile
Solvent B :0.1%Trifluoroacetic in 100% Water

Gradient :	A	B
0.01min	30%	70%
25in	55%	45%
25.1min	100%	0%
30min	Stop	

Flow rate :1.0ml/min
Wavelength :220nm
Volume: 10μl

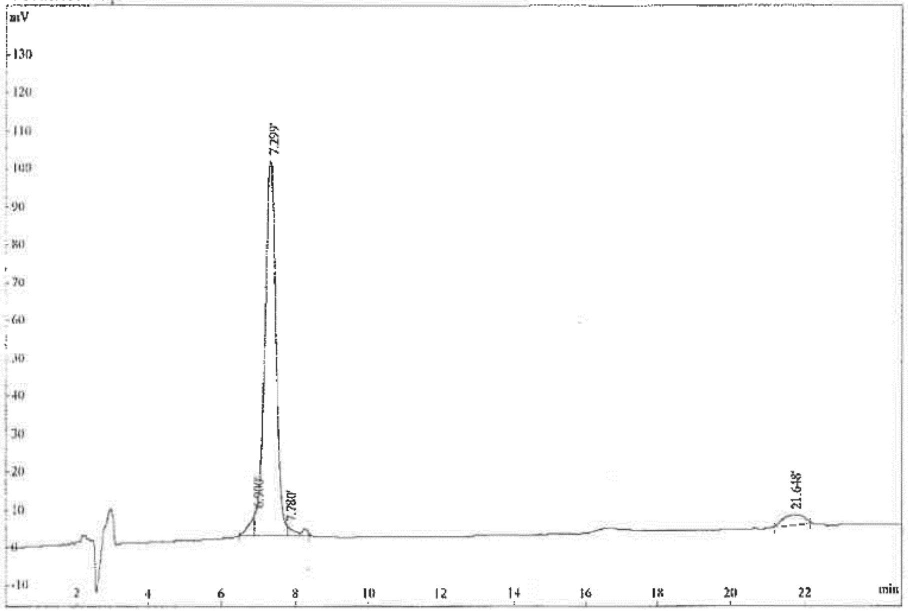

Cecropin A

Column :4.6*250mm, phenomenex C18-5
Solvent A :0.1%Trifluoroacetic in 100% Acetonitrile
Solvent B :0.1%Trifluoroacetic in 100% Water

Gradient :	A	B
0.01min	26%	74%
25in	51%	49%
25.1min	100%	0%
30min	Stop	

Flow rate :1.0ml/min
Wavelength :220nm
Volume: 10μl

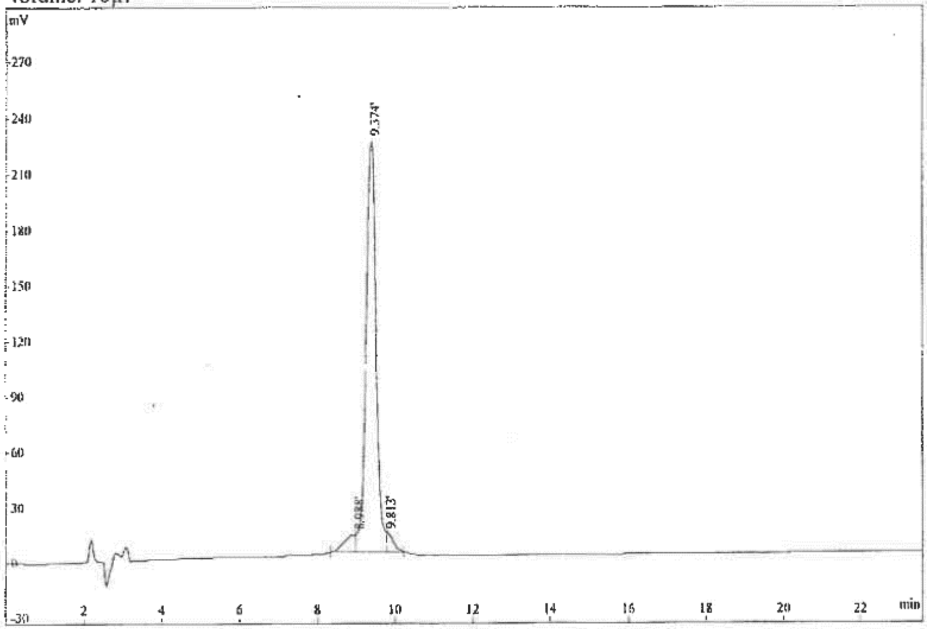

Sarcotoxin IA

Column :4.6*250mm, phenomenex C18-5
Solvent A :0.1%Trifluoroacetic in 100% Acetonitrile
Solvent B :0.1%Trifluoroacetic in 100% Water

Gradient :	A	B
0.01min	30%	70%
25in	55%	45%
25.1min	100%	0%
30min	Stop	

Flow rate :1.0ml/min
Wavelength :220nm
Volume: 10μl

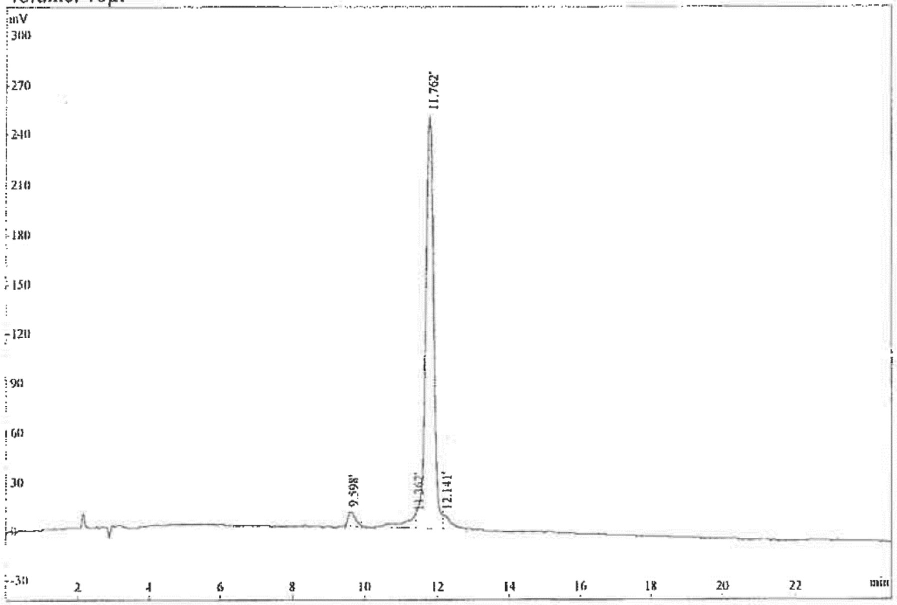

Cecropin A

Column :4.6*250mm, phenomenex C18-5
Solvent A :0.1%Trifluoroacetic in 100% Acetonitrile
Solvent B :0.1%Trifluoroacetic in 100% Water

Gradient :	A	B
0.01min	32%	68%
25in	57%	43%
25.1min	100%	0%
30min	Stop	

Flow rate :1.0ml/min
Wavelength :220nm
Volume: 10μl

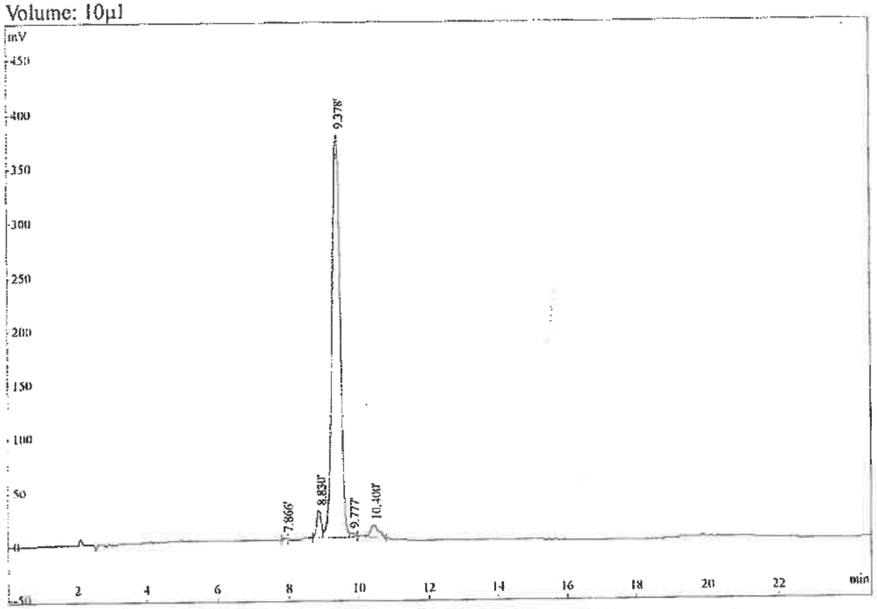

Ceratotoxin

Column :4.6*250mm, phenomenex C18-5
Solvent A :0.1%Trifluoroacetic in 100% Acetonitrile
Solvent B :0.1%Trifluoroacetic in 100% Water

Gradient :	A	B
0.01min	25%	75%
25in	50%	50%
25.1min	100%	0%
30min	Stop	

Flow rate :1.0ml/min
Wavelength :220nm
Volume: 10μl

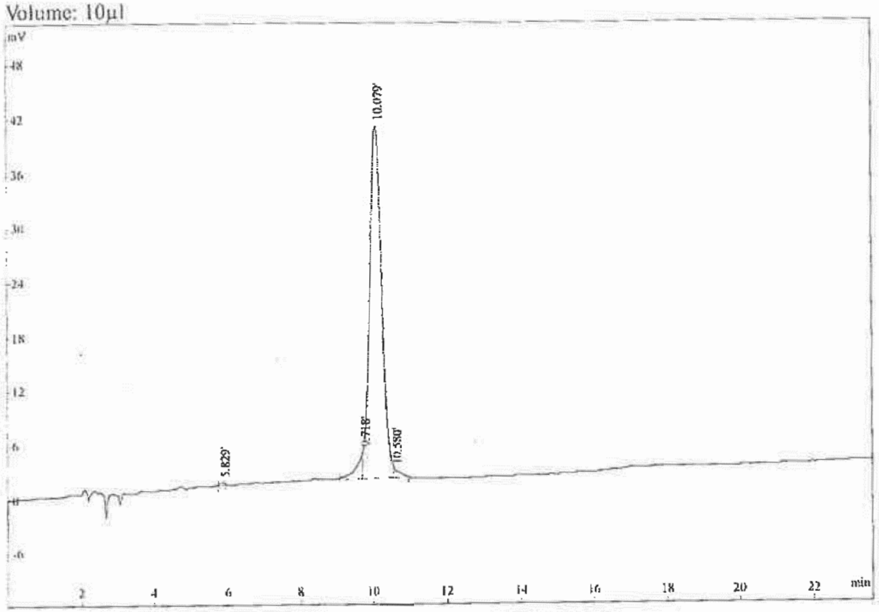

Stomoxyn

Column : 4.6*250mm, Welch CX-C18 5um
Solvent A : 0.1% trifluoroacetic in 100% acetonitrile
Solvent B : 0.1% trifluoroacetic in 100% water

Gradient	A	B
0.01min	22%	78%
25.00min	47%	53%
25.10min	100%	0%
30.0min	STOP	

Flow rate : 1.0ml/min
Wavelength : 220nm
Volume : 10ul

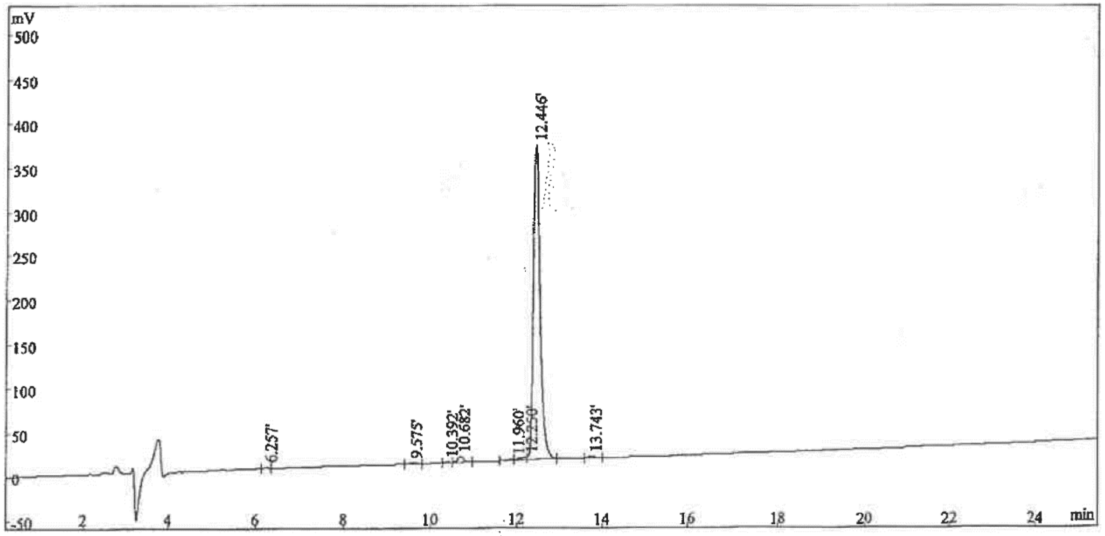

Spinigerin

Column : 4.6×250mm, Venusil MP C18-5
Solvent A : 0.1% trifluoroacetic in 100% acetonitrile
Solvent B : 0.1% trifluoroacetic in 100% water
Gradient :

	A	B
0.01min	16%	84%
25min	41%	59%
25.1min	100%	0%
30min	STOP	

Flow rate : 1.0 mL/min
Wavelength : 220nm
Volume : 5ul

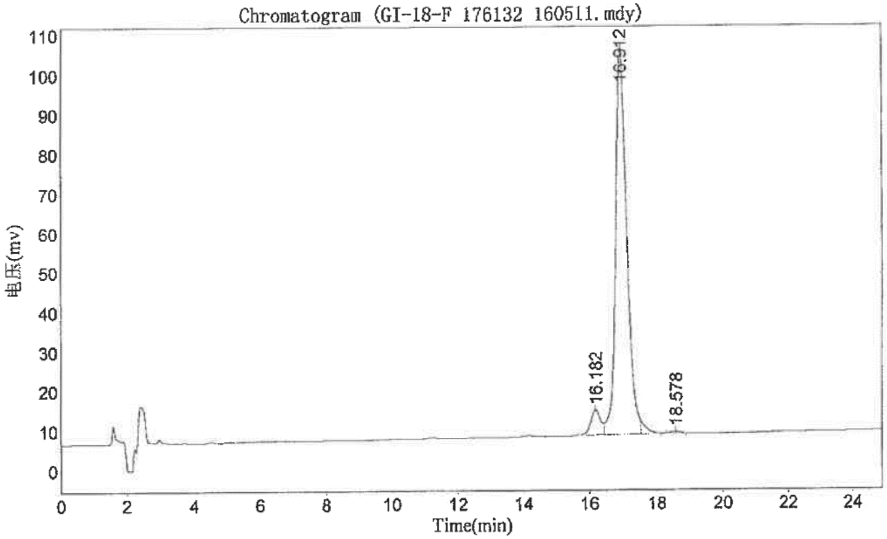

Apidaecin Ia

Column : 4.6×250mm, Venusil MP C18-5
Solvent A : 0.1% trifluoroacetic in 100% acetonitrile
Solvent B : 0.1% trifluoroacetic in 100% water
Gradient :

	A	B
0.01min	16%	84%
25min	41%	59%
25.1min	100%	0%
30min	STOP	

Flow rate : 1.0 mL/min
Wavelength : 220nm
Volume : 5ul

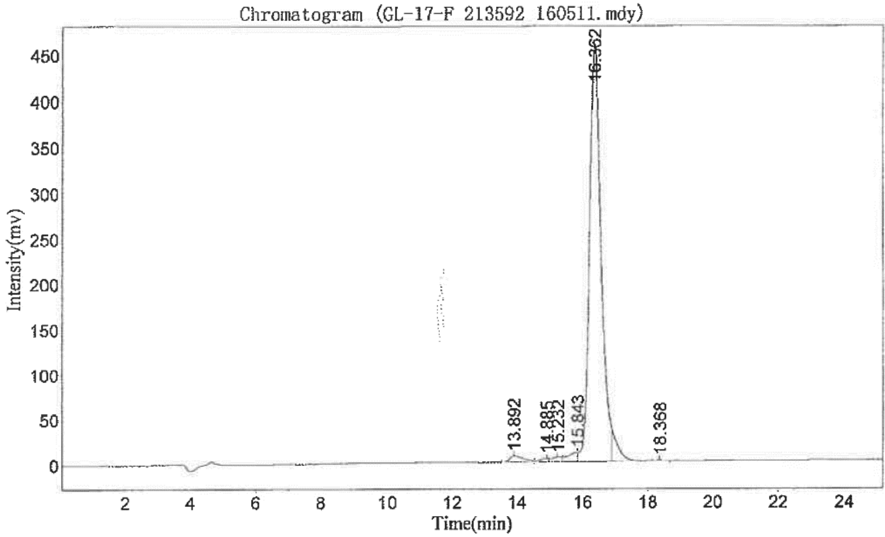

Apidaecin

Column : 4.6×250mm, Venusil MP C18-5
Solvent A : 0.1% trifluoroacetic in 100% acetonitrile
Solvent B : 0.1% trifluoroacetic in 100% water
Gradient :

	A	B
0.01min	12%	88%
25min	37%	63%
25.1min	100%	0%
30min	STOP	

Flow rate : 1.0 mL/min
Wavelength : 220nm
Volume : 5ul

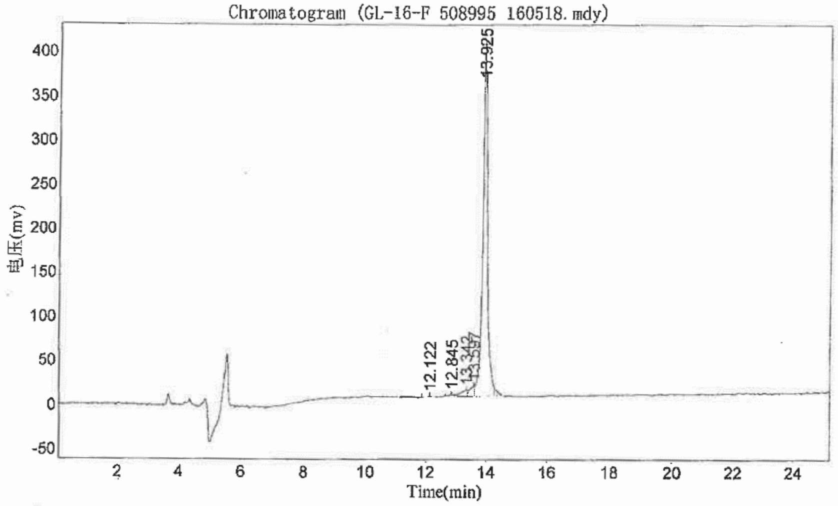

Formaecin-1

Column : 4.6×250mm, Venusil MP C18-5
Solvent A : 0.1% trifluoroacetic in 100% acetonitrile
Solvent B : 0.1% trifluoroacetic in 100% water
Gradient :

	A	B
0.01min	10%	90%
25min	35%	65%
25.1min	100%	0%
30min	STOP	

Flow rate : 1.0 mL/min
Wavelength : 220nm
Volume : 5ul

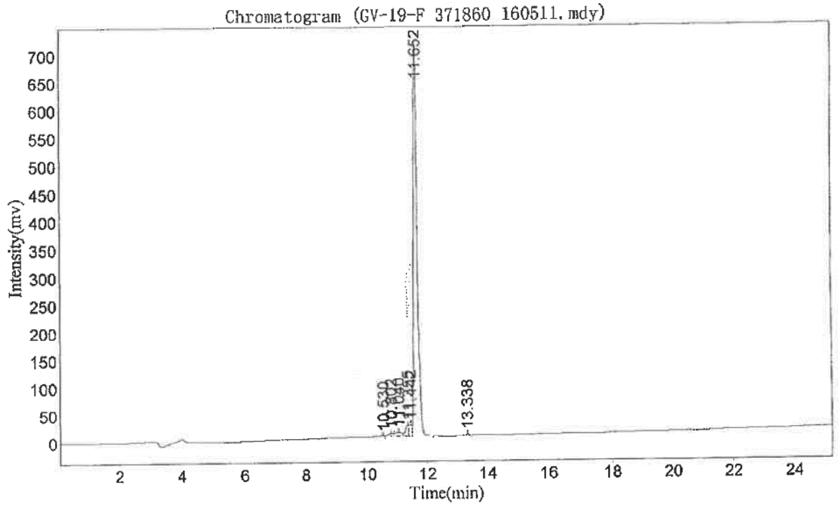

Drosocin

Column : 4.6×250mm, Venusil MP C18-5
Solvent A : 0.1% trifluoroacetic in 100% acetonitrile
Solvent B : 0.1% trifluoroacetic in 100% water
Gradient :

	A	B
0.01min	12%	88%
25min	38%	62%
25.1min	100%	0%
30min	STOP	

Flow rate : 1.0 mL/min
Wavelength : 220nm
Volume : 5ul

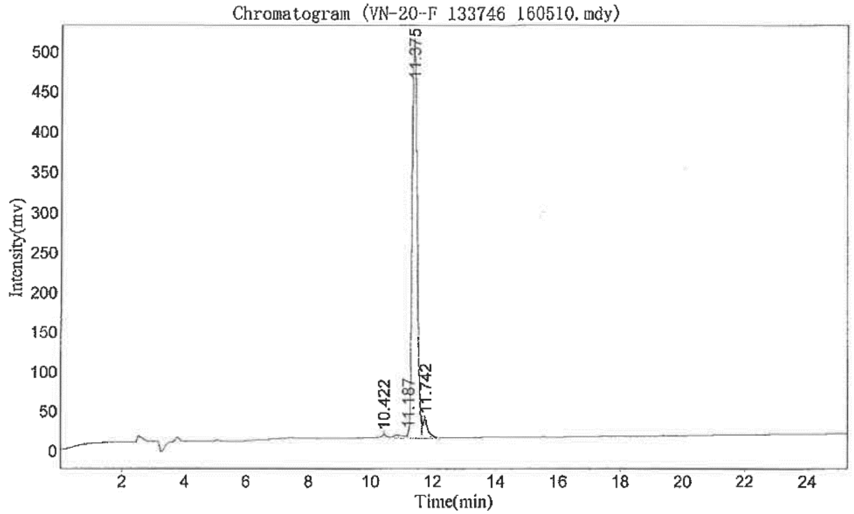

Pyrrhocoricin

Column : 4.6×250mm, Venusil MP C18-5
Solvent A : 0.1% trifluoroacetic in 100% acetonitrile
Solvent B : 0.1% trifluoroacetic in 100% water
Gradient :

	A	B
0.01min	11%	89%
25min	36%	64%
25.1min	100%	0%
30min	STOP	

Flow rate : 1.0 mL/min
Wavelength : 220nm
Volume : 5ul

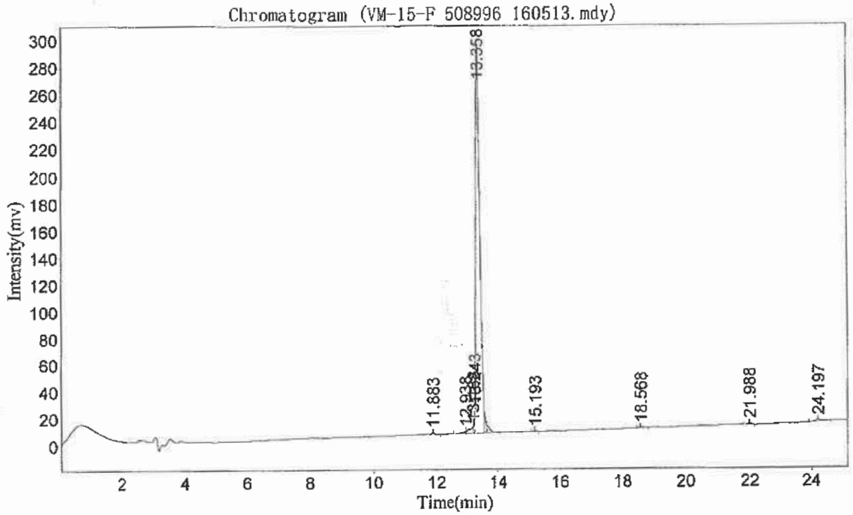

Metalnikowin-1

[illegible]
Column : 4.6×250mm, Venusil MP C18-5
Solvent A : 0.1% trifluoroacetic in 100% acetonitrile
Solvent B : 0.1% trifluoroacetic in 100% water
Gradient :

	A	B
0.01min	11%	89%
25min	36%	64%
25.1min	100%	0%
30min	STOP	

Flow rate : 1.0 mL/min
Wavelength : 220nm
Volume : 5ul

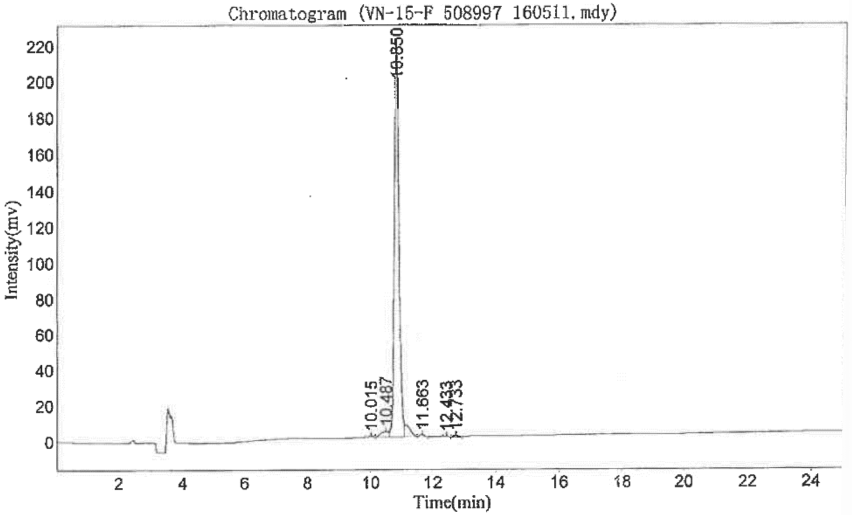

Metalnikowin-1

Lot No : [illegible]
Column : 4.6×250mm, Venusil MP C18-5
Solvent A : 0.1% trifluoroacetic in 100% acetonitrile
Solvent B : 0.1% trifluoroacetic in 100% water
Gradient :

	A	B
0.01min	21%	79%
25min	46%	54%
25.1min	100%	0%
30min	STOP	

Flow rate : 1.0 mL/min
Wavelength : 220nm
Volume : 5ul

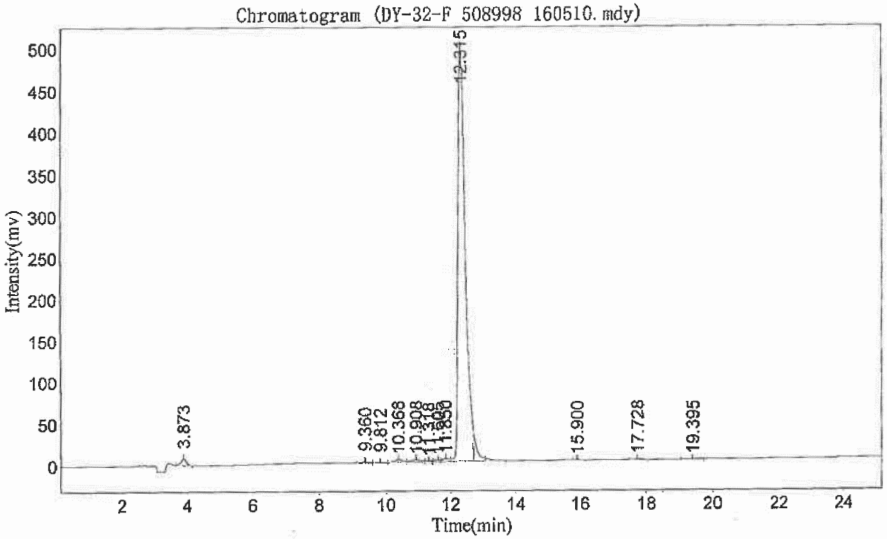

Metalnikowin-2A

Column	:	4.6*250mm, Welch CX-C18 5um
Solvent A	:	0.1% trifluoroacetic in 100% acetonitrile
Solvent B	:	0.1% trifluoroacetic in 100% water

Gradient

	A	B
0.01min	20%	80%
25.00min	45%	55%
25.10min	100%	0%
30.0min	STOP	

Flow rate	:	1.0ml/min
Wavelength	:	220nm
Volume	:	10ul

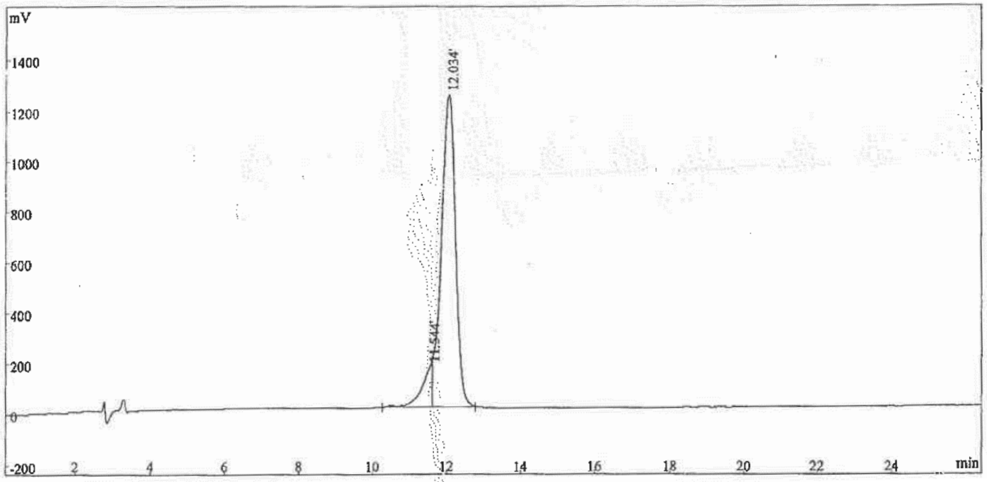

Abaecin

Column	:	4.6*250mm, Welch CX-C18 5um
Solvent A	:	0.1% trifluoroacetic in 100% acetonitrile
Solvent B	:	0.1% trifluoroacetic in 100% water

Gradient

	A	B
0.01min	16%	84%
25.00min	41%	59%
25.10min	100%	0%
30.0min	STOP	

Flow rate	:	1.0ml/min
Wavelength	:	220nm
Volume	:	10ul

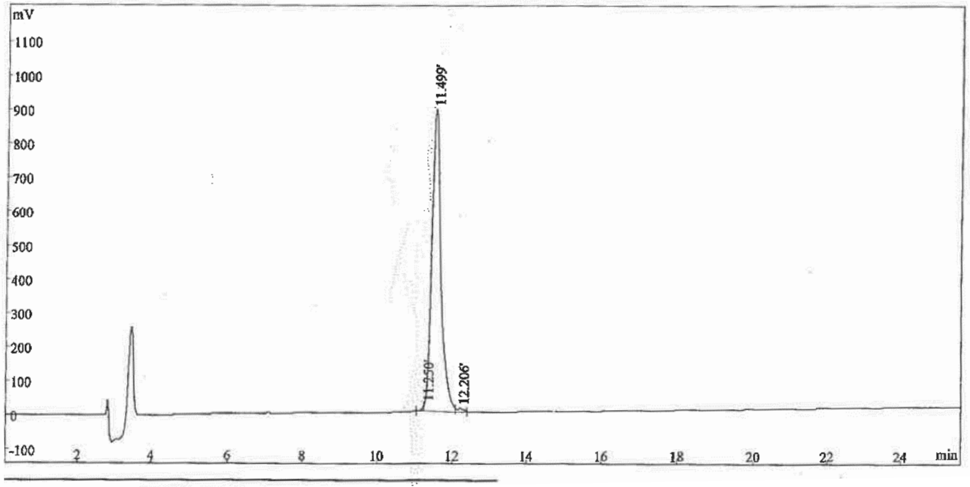

Metchnikowin-1

Column	:	4.6*250mm, Welch CX-C18 5um
Solvent A	:	0.1% trifluoroacetic in 100% acetonitrile
Solvent B	:	0.1% trifluoroacetic in 100% water

Gradient

	A	B
0.01min	16%	84%
25.00min	41%	59%
25.10min	100%	0%
30.0min	STOP	

Flow rate	:	1.0ml/min
Wavelength	:	220nm
Volume	:	10ul

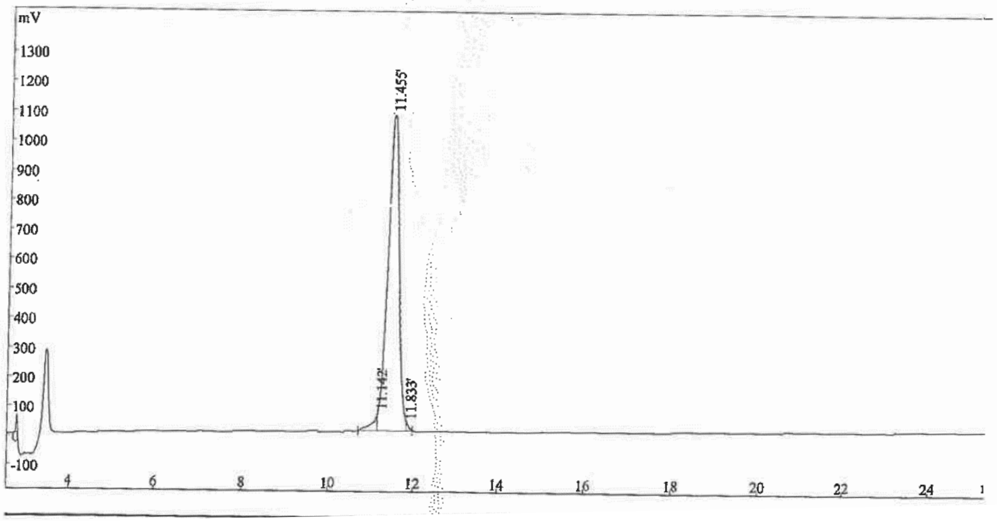

Metchnikowin-2

Lot. No [illegible]

Pump A	:0.1% trifluoroacetic in 100% water
Pump B	:0.1% trifluoroacetic in 100% acetonrtrile
Total Flow	:1ml/min
Wavelength	:214nm
Analytical column type	:SHIMADZU Inertsil ODS-SP(4.6*250mm*5um)
Dissolution method	:15%ACN+85%H2O
Acquisition Time	:2017/7/4 17:32:39
Inj. Volume	:95ul

Time	Module	Action	Value
0.01	Pumps	B.Conc	2
30.00	Pumps	B.Conc	65
33.00	Pumps	B.Conc	100
38.00	Pumps	B.Conc	100
40.00	Pumps	B.Conc	2
50.00	Controller	Stop	

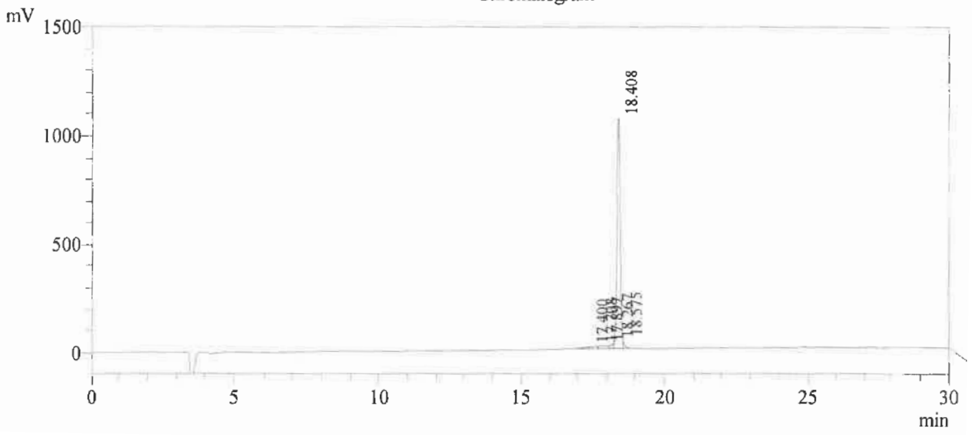

Defensin Tca1

Column	: 4.6×250mm,Baston Green C4 300A		
Solvent A	: 0.1% trifluoroacetic in 100% acetonitrile		
Solvent B	: 0.1% trifluoroacetic in 100% water		
Gradient		A	B
	0.01min	15%	85%
	25.00min	40%	60 %
	25.10min	100%	0%
	30.0min	STOP	
Flow rate	: 1.0ml/min		
Wavelength	: 220nm		
Volume	: 10ul		

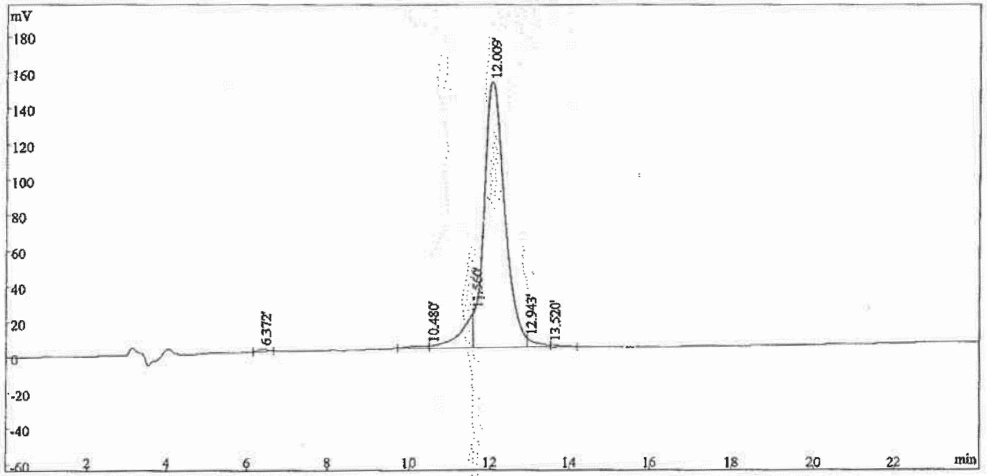

Cecropin-like

Column	: 4.6*250mm, Welch CX-C18 5um		
Solvent A	: 0.1% trifluoroacetic in 100% acetonitrile		
Solvent B	: 0.1% trifluoroacetic in 100% water		
Gradient		A	B
	0.01min	20%	80%
	25.00min	45%	55%
	25.10min	100%	0%
	30.0min	STOP	
Flow rate	: 1.0ml/min		
Wavelength	: 220nm		
Volume	: 10ul		

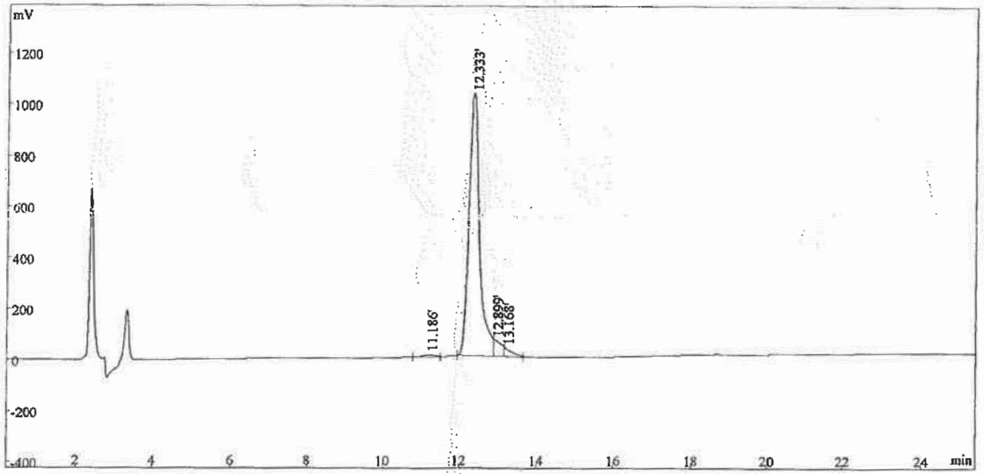

Cecropin

Column	: 4.6*250mm, Welch CX-C18 5um		
Solvent A	: 0.1% trifluoroacetic in 100% acetonitrile		
Solvent B	: 0.1% trifluoroacetic in 100% water		
Gradient		A	B
	0.01min	21%	79%
	25.00min	46%	54%
	25.10min	100%	0%
	30.0min	STOP	
Flow rate	: 1.0ml/min		
Wavelength	: 220nm		
Volume	: 10ul		

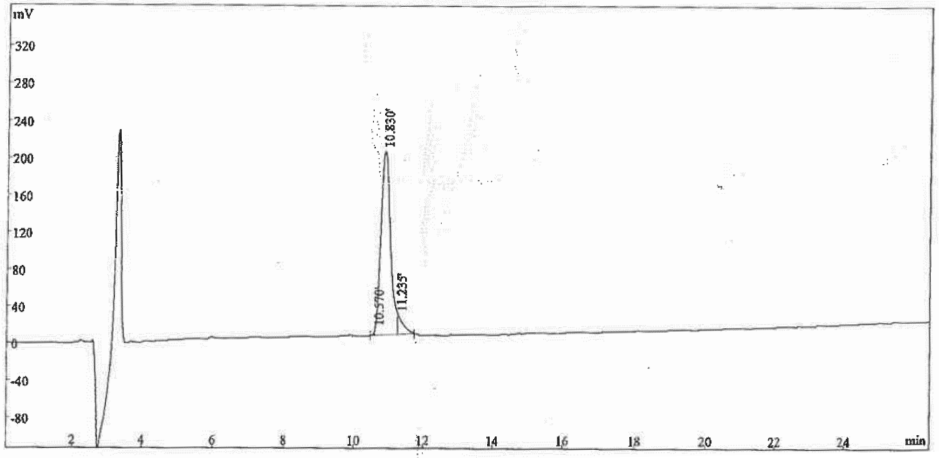

Cecropin (Sarcotoxin 1C)

Column	: 4.6*250mm, Welch CX-C18 5um		
Solvent A	: 0.1% trifluoroacetic in 100% acetonitrile		
Solvent B	: 0.1% trifluoroacetic in 100% water		
Gradient		A	B
	0.01min	20%	80%
	25.00min	45%	55%
	25.10min	100%	0%
	30.0min	STOP	
Flow rate	: 1.0ml/min		
Wavelength	: 220nm		
Volume	: 10ul		

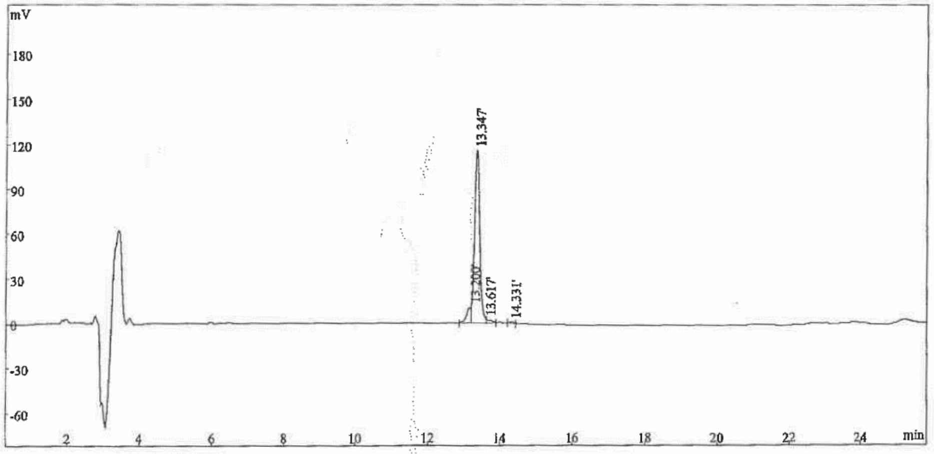

Stomoxyn

Column : 4.6*250mm, Welch CX-C18 5um

Solvent A : 0.1% trifluoroacetic in 100% acetonitrile

Solvent B : 0.1% trifluoroacetic in 100% water

Gradient

	A	B
0.01min	25%	75%
25.00min	50%	50 %
25.10min	100%	0%
30.0min	STOP	

Flow rate : 1.0ml/min

Wavelength : 220nm

Volume : 10ul

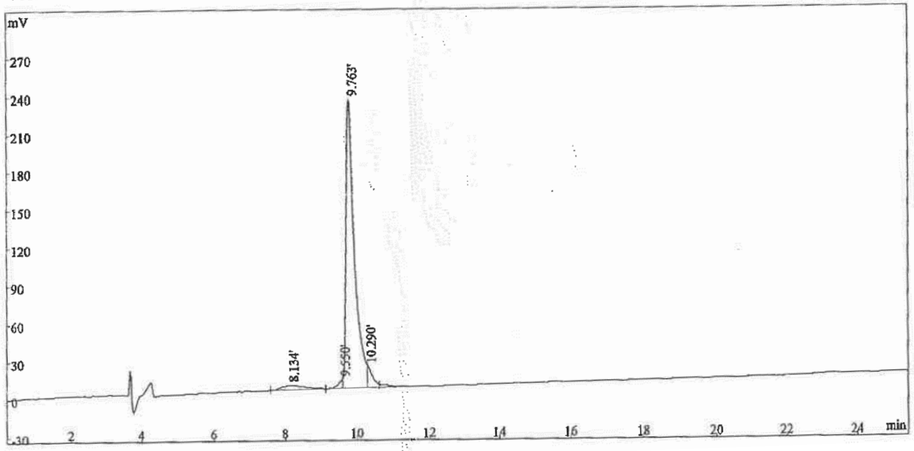

Cecropin (Sarcotoxin)
